# Dissemination or participation? Exploring scientists’ definitions and science communication goals in the Netherlands

**DOI:** 10.1371/journal.pone.0277677

**Published:** 2022-12-01

**Authors:** Adina Nerghes, Bob Mulder, Ju-Sung Lee

**Affiliations:** 1 Strategic Communication, Department of Social Sciences, Wageningen University & Research, Wageningen, The Netherlands; 2 Philosophy, Department of Social Sciences, Wageningen University & Research, Wageningen, The Netherlands; 3 Erasmus School of History, Culture and Communication, Department of Media and Communication, Erasmus University Rotterdam, Rotterdam, The Netherlands; University of Exeter, UNITED KINGDOM

## Abstract

The field of science communication has grown considerably over the past decade, and so have the number of scientific writings on what science communication is and how it should be practiced. The multitude of theoretisations and models has led to a lack of clarity in defining science communication, and to a highly popularised—and theorised—rhetorical shift from deficit to dialogue and participation. With this study, we aim to remediate the absence of research into what science communication is, for scientists themselves. We also investigate whether the transition towards dialogue and participation is reflected in the goals scientists identify as important to their science communication efforts, both in a general and a social media context. For this, we analyse survey data collected from scientists in the Netherlands using thematic qualitative analysis and statistical analysis. Our results reveal six main dimensions of science communication as defined by our respondents. The 584 definitions we analyse demonstrate a focus on a one-way process of transmission and translation of scientific results and their impacts towards a lay audience, via mostly traditional media channels, with the goals of making science more accessible, of educating audiences, and of raising awareness about science. In terms of the goals identified as most important by scientists in the Netherlands, we find goals aligned with the deficit and dialogue models of science communication to be the most important. Overall, our findings suggest we should be cautious in the face of recent claims that we live in a new era of dialogue, transparency, and participation in the realm of science communication.

## Introduction

In recent years, science communication has received increasing attention from politics and society, and it has developed into a vital part of academic activity [1]. As science communication is of rising importance for scientists, and even for their academic careers [2], our work aims to bring a timely and important contribution to this area of research. We aim to contribute towards a broader understanding and deeper insights into science communication from the perspective of scientists in terms of individual viewpoints and the goals scientists focus on in their science communication practices as well as the goals scientists are trying to achieve when using social media for science communication.

As a growing area of practice and research, science communication over the last 50 years has developed into a field primarily concerned with answering questions related to science and society, science in the media, and the role of science journalists [3]. In the academic realm, specifically, these questions are addressed by researchers of sociology, journalism, and media studies. As it is the case with most multidisciplinary, rich research fields, science communication has produced different—and sometimes even contradictory—theorisations [4]. It comes as no surprise, then, that scholars have not agreed on a clear and shared definition of science communication [4, 5].

In very general terms, science communication refers to communication on scientific topics and the relationship between science and society. But as Burns, O’Connor and Stocklmayer state [6], the definition of science communication has been “plagued by an unfortunate lack of clarity” (p.183). The wide array of definitions offered by the science communication literature ranges from those referencing some set of signature methods and aims [7] to those focusing on “manifest or latent *purposes* of science communication practice” [4, p.4].

For example, Davies [8] defines science communication in a contemporary conception as “organized processes that seek to engage lay publics with scientific knowledge.” (p.163) While this definition seems simple and straightforward, it restricts science communication to those processes in which the public engages with scientific knowledge as a finished product. Such definitions are in clear contrast to those highlighting the importance of public engagement in the process of generating scientific knowledge [9, 10] and those advocating for a change in perspective—from a communication process to a ‘conversation’ [4]. Bryant [11] takes further steps to acknowledge the intangible cultural aspects of science communication and that science communication is a continual process rather than a one-off, linear activity and defines science communication as “… the processes by which the culture and knowledge of science are absorbed into the culture of the wider community.”

Scholars such as Burns [6] take a more pragmatic approach, formulating a definition aimed at providing the framework for evaluating the effectiveness of science communication from an outcomes oriented perspective:

“Science communication […] is defined as the use of appropriate skills, media, activities, and dialogue to produce one or more of the following personal responses to science (the AEIOU vowel analogy): Awareness, Enjoyment, Interest, Opinion-forming, and Understanding.”

The definitions exemplified above are by no means the only ones nor do they include all involved actors, beyond scientists and the lay public (such as institutions, journalists, etc.) and all societal contexts (e.g., policy-making). However, an exhaustive review of all the science communication definitions is beyond the scope of this article. Rather than attempt to further portray the breadth of science communication definitions, we would like to highlight the importance of exploring scientists’ personal science communication definitions. While some studies have attempted to understand scientists’ choices and objectives using the Theory of Planned Behavior [12] and the associated Integrated Behavioral Model [13], and others have focused on understanding scientists’ views about specific communication objectives and strategies [14], our contribution focuses on uncovering how scientists themselves define science communication.

A survey conducted in the UK by Illingworth and colleagues [15] further highlights the importance of understanding scientists’ personal definitions of science communication and how such definitions may be more nuanced and expansive than those presented by literature or various organisations. They show that, even though scientists believed their personal definitions were aligned with pre-existing ones, in reality the very wide range of personal definitions was more nuanced and expansive [15]. Thus, empirically assessing various aspects of science communication from the perspective of a pre-determined definition creates an unnecessarily rigid analysis framework.

Empirical assessment departing from the scientists’ personal science communication definitions can foster a broader understanding and deeper insights [16, 17] into individual viewpoints [18, 19]. By focusing on scientists’ “logic and the kinds of evidence they considered worthwhile and relevant, we suspend our own notions of what is right.” [20] In other words, the investigation of personal definitions can help us identify and understand what science communication is from the perspective of those who are closest to the scene. Here we argue that—while understudied—scientists’ personal definitions form the basis for their approaches to science communication and their communication activities. The importance of understanding scientists’ “individual conceptions of science communication” (or what the authors refer to as ‘mental models’) is further highlighted by a very recent study conducted by Kessler and colleagues across three European countries [21]. Their findings suggest that scientists’ individual conceptions largely align with the ways in which they practice science communication.

The scientific community has often been primarily focused on disseminating information and building public knowledge about science [22–24] and on developing theoretical models that describe the relationship between scientists and the public [25, 26]. However, very few studies have focused on understanding the specific goals scientists are trying to accomplish through their science communication efforts [21, 27], and scholars have generally not treated communication choices related to strategies, objectives, and goals as behaviours that can both be studied and reshaped [28].

Over the last decades, a rhetorical shift has taken place in the area of science communication in that calls for dialogue between scientists and non-scientists, as well as calls for more participatory approaches to science communication, have taken precedence over the scientific literacy deficits rhetoric. Together with this rhetorical shift from ‘deficit’ towards ‘dialogue’ and ‘participation’, scholars have developed a number of science communication models ranging from transmission of information to dialogue and public engagement. Such models are said to be frameworks for understanding what the “problem” is, how to measure the problem, and how to address the problem [29, p.13]. Models of science communication, emerging in the literature, represent how theorists believe that science has been, is being, or should be communicated [30]. However, as we will argue, the assumptions behind most of these models have not been comprehensively tested against real-life practices of science communication [31]. But let us first discuss three of the most prominent models, their underlining assumptions, and described goals.

Historically, science communication had been described as a process of information transmission, which assumed “public deficiency, but scientific sufficiency” [32]. What has become known as the deficit model of science communication was first discussed in the literature during the 1980s and early 1990s [33, 34]. This model asserts that science communication is guided by a perceived need for science literacy, where the public is viewed as having a deficit of scientific knowledge until such knowledge is received from the communicator [35–37]. As Irwin [38] describes, the deficit model aims at ‘public understanding of science’ and is characterised as a one-way, top-down communication process. In other words, science communication is a one-way process from the scientists (‘experts’) to the public (‘non-experts’) in which scientists—with all the required information—filled the knowledge vacuum in the scientifically illiterate general public as they see fit [39].

The perspective of the deficit model is perfectly exemplified by the UK Chief Scientific Adviser to the House of Lords, in a report published in 2000 which purports “difficulties in the relationship between science and society are due entirely to ignorance on the part of the public” and “with enough public-understanding activity, the public can be brought to greater knowledge, whereupon all will be well.” [40, p.25]. This assumption that by fixing the deficit everything will be ‘well’ has led to the emergence of many large-scale projects focused on science literacy, often addressing revisions of national educational curricula [41]. However, research has shown not only that the deficit assumption is an incorrect one—as people with limited education can quickly come to understand highly complex technical information [42]—but that after more than 25 years of ‘filling the deficit,’ the public’s ‘knowledge’ seems unaffected and remarkably stable [43]. Under the deficit perspective, by making the knowledge deficits of the public central to the problem of science communication, many scientists ignore the possibility that their communication approaches might be part of the problem [44].

Scholars have theorised that the condescending claims of the public’s ignorance under the deficit model [45] and the increasing signs of public unease with science during the 1980s [30] have led to a new style of science communication as embodied in the dialogue model. This new model of science communication draws on the belief that “more active, open and democratic relations between science and citizens are both desirable and necessary.” [38, p.200]

The dialogue model is characterised by three main features: (1) engagement in a dialogue with the public to help explain the science [46], (2) listening to and consulting the public about their perceptions, concerns and needs with regard to science [37, 47], and (3) acknowledging that the public may have useful knowledge that can help scientific progress and policy-making [48]. Under this model, science communication is centred around dialogic approaches that aim for a two-way conversation, allowing an exchange of perspectives between scientists and the public.

The emergence of the dialogue model, together with the increasing demand for scientists’ involvement in public discussions and with policy documents shifting their language from ‘communication’ to ‘dialogue,’ have created a powerful narrative in which this type of science communication is portrayed as inherently superior [49]. However, many scholars have argued that this shift from deficit to dialogue has not been as complete as the narrative suggests [37]. More than ten years of research evidence has questioned the scale of this supposed shift and the extent to which the dialogue model goals and principles have been adopted in actual science communication practices [46, 50–54]. The apparent replacement of the deficit model, as Wynne [46] concludes, is more nominal than real. Thus, even though the science communication vocabulary has changed considerably over time, the underlying assumptions may still be inline with those that inform a deficit model perspective [37].

In recent years, the narrative shift has evolved even further to include public participation or public engagement. Public participation (or public engagement) models have emerged as a direct attempt to enhance social trust in science policy. These models focus on a series of activities—consensus conferences, citizen juries, deliberative technology assessments, science shops, deliberative polling, etc. [55, 56]—driven by a commitment to ‘democratize science’ through some form of empowerment and political engagement of the public. Although rarely linked in science communication literature, the public participation model shares many similarities to more established techniques, such as public meetings and public hearings [57, 58].

Participation models signal a more obvious shift in power than the dialogue model and emphasise the role of the public and other societal stakeholders in reflecting upon, sharing knowledge about, creating new knowledge, and making decisions about science that affects society [9, 10]. Participation models seek to set the sciences in a wider social context, addressing societal concerns and priorities that involve multiple stakeholder perspectives [38]. It is argued that such approaches to science communication are driven by the intent of ‘democratizing’ science by giving the public more control over science through some form of empowerment and political engagement [59].

Scholars have posed a number of criticisms related to participation models due to their focus on the process of science over any substantive content, their limitations in terms of the numbers of people they serve, and for sometimes exhibiting an ‘anti-science’ bias given their focus on lay/local knowledge over scientific knowledge [41]. The strongest criticism, however, relates to the fact that such models are said to address politics more than the public understanding of science due to their commitment to a particular stance about political relations [60, 61].

The three science communication models discussed above, driven by different assumptions, provide only schematic tools for theorising about science communication activities. While the deficit model centres around filling a knowledge vacuum and the dialogue model on an exchange of perspectives between the sciences and the public, the participatory model centres on the ‘democratisation of science’ through some form of empowerment and political engagement of the public. The emergence of these models (and many others) together with the strong rhetorical shift from deficit to dialogue and participation—both in scholarly and policy discourses—seem to indicate a unidirectional transition process. However, Bucchi and Trench [4] suggest that rather than looking at a ‘fixed triad of deficit, dialogue and participation’ (p.8), this range of models should be seen as a dynamic spectrum that is continually growing in both directions.

While the radical transition—from deficit to dialogue and participation—and the concurrent rhetorical shift have been deemed highly implausible over such a short period of time [37, 46], very few studies have endeavoured to empirically assess the specific goals scientists are trying to accomplish through their science communication efforts [27] and how their goals align with those of the deficit, dialogue, and participation science communication models. Although research has shown that many scientists accept and support dialog and participation as some kind of ‘gold standards,’ or are at least are aware of the push toward public communication [62], not many studies focused on assessing how this translates into actual science communication practices. One of the few studies investigating such practices found that—due to the continuing adoption of a simplistic contrast structure that opposes science and the public as two self-contained, antagonistic social entities—the shift towards more democratic engagement of the public has not been as profound and complete as expected [63].

In this article, we aim to address this research gap by investigating the goals scientists focus on in their science communication practices and the alignment of these goals with the three models discussed thus far. To do so, we build on the work of Metcalfe [30] that lists the primary goals inherent to each of the three science communication models, based on a thorough literature review. We further discuss these goals and their importance to our work in our Data and Methods section.

When discussing contemporary science communication practices, one must not omit to highlight the radical and important changes brought about by the emergence of social media channels. On a daily basis, millions of people all over the world are constantly consuming and creating content through social media platforms. Considering the popularity of such platforms (e.g., 192 million daily active users on Twitter), it is easy to see that information disseminated through these channels can reach millions in a matter of minutes and, as Van Eperen and Marincola [64] acknowledge, that successful communication can only be achieved by using the channels in which the public is currently engaged. Thus, social media channels offer a powerful venue through which scientists can act as a public voice for science in a quick and efficient manner by using a medium in which the public is already engaged [65].

Beyond reaching engaged and diverse publics—researchers, the general public, government, and all other stakeholders—social media platforms have the potential to enable multi-vocal, multi-way communication [39]. Such platforms are said to “facilitate interactive information sharing” [66] and to increase the depth and reach of engagement among stakeholders [67]. The potential of social media platforms to involve a wide variety of stakeholders in an interactional manner have long been emphasised by proponents of the dialogue and participation science communication models [39, 68]. However, in reality, many researchers are cautious in changing traditional scholarly communication patterns in response to social media [69] and the area of online science communication remains understudied [65].

When it comes to science communication, although social media seems to be the ideal environment for two-way and multi-vocal communication models, the few studies that have investigated online science communication have found that this is only one part of the story. science communication practitioners and science organisations use social media for one-way message dissemination more often than they truly engage with their publics [70, 71], and they generally underutilise social media’s potential to create true dialogue with their audiences [72]. For instance, when conducting a review of social media activities of 11 US based science agencies, Lee and VanDyke [73] found that their outreach activities did not facilitate two-way interactions very well, suggesting an adherence to a deficit-model way of thinking for these agencies. Similarly, analysing Twitter utilisation patterns of NanoDays science festivals organised between 2012 to 2015, Su and colleagues [74] found that most tweets exemplified a one-way, information-sharing model of communication.

Just as in the case of the proposed radical transition of science communication—from deficit to dialogue and participation—the belief that social media affordances foster unprecedented opportunities for two-way and multi-vocal science communication is becoming more or less an accepted fact. But, as the few studies that have investigated social media use for science communication have shown [73–75], one-way, information-sharing practices aligned with the deficit model still dominate. So if social media offers such opportunities, how come scientists still engage in information transmission behaviours? Here, we argue that in order to answer this question, we need to first have a better understanding of the goals scientists are trying to achieve through their social media science communication practices. To bring a contribution to this under-researched area of science communication, we investigate not only the goals scientists consider as most important in the context of general science communication, but we also assess which goals they find important in the context of social media science communication. To do this, we used a modified version of Metcalfe’s [30] list, specifically reworded for social media. We will further discuss this list in our Materials and methods section.

Considering the promises of social media as a participatory environment where already-engaged audiences can easily be reached, we also aim to investigate whether those actively using social media for their science communication efforts may approach science communication from a more participatory oriented perspective. To do so, we aim to compare between social media users and non-users and their general science communication goals.

### Aims

In sum, the aims of our work are four fold. First, we aim to contribute to a broader understanding and deeper insights into what science communication is, beyond conceptual definitions proposed by literature. Moving past the unnecessarily narrow and rigid science communication definitions proposed by various scholars, we aim to grasp the perspectives of some of the core actors in the field, scientists themselves. Second, we set to investigate whether the highly popularised—and theorised—rhetorical shift from deficit to participation is reflected in the goals scientists identify as important to their science communication efforts. In other words, we aim to understand the scientists’ goals and which of the models are at work in their science communication activities. Third, we aim to provide a stepping stone towards a better understanding of why a deficit-model way of thinking remains dominant in social media science communication efforts. Lastly, we aim to uncover whether those actively using social media for their science communication efforts may approach science communication from a more dialogue or participatory oriented perspective, rather than the deficit perspective shown by previous studies.

## Materials and methods

### Ethics statement

The survey data for this study were collected in accordance to the guidelines of the Association of Universities The Netherlands [76] and ethical approval was not required. Under Dutch law, ethical approval is not required for conducting survey questionnaire research in the Netherlands when such surveys do not target children or other vulnerable groups and they do not address confidential or sensitive issues [77]. Furthermore, ethical approval for this study was deemed unnecessary by the check-list provided by the Social Sciences Ethics Committee of Wageningen University & Research.

Participants in our survey gave informed consent before their answers were recorded. The first page of our survey provided a full explanation of the scope and aims of our study and it informed respondents that participation is anonymous and voluntary. Furthermore, at the end of the survey, respondents were informed that by clicking the submit button they consent for their answers to be included in our study and that they could withdraw from the study at any time. To ensure the anonymity of the respondents, personal identifiers such as name, e-mail address, physical address, and organization name were not collected. Our respondents had the opportunity to provide us with their social media information (e.g., Twitter handle) for a later stage of this project and to leave an email address for a chance at winning a gift card. Their social media information and email addresses are stored separately from their survey responses and will not be included in any reports using these survey data.

### Survey data

The data included in this study comes from a large scale survey we conducted in the Netherlands between April 1st and May 31st 2021. The survey was specifically addressed to researchers, at any career level, working in any public research or technical university or research institute in the Netherlands.

Our survey was disseminated via multiple channels in an attempt to collect a representative sample for researchers in the Netherlands. Invitations to participate, which included an anonymous link or a QR-code, were placed in several university-based magazines (online and in print), newsletters, and intranet pages. We also posted similar invitations on Twitter, Facebook, and LinkedIn. Lastly, for every university in the Netherlands, the survey was disseminated via email, based on an email address list generated using the public online directories provided on each university’s websites, for each of their faculties. Alongside the universities, five publicly funded research institutes were also targeted.

While our email address list included over 20,000 emails, it did not capture those scientists whose contact information may not have been provided online or was not updated in the university contact directories. In order to limit inconveniencing our potential respondents, no reminder emails were sent after the first invitation email. Of the total of 584 respondents who completed our survey, 2 of our respondents accessed the survey via the QR-code, 10 via social media links, and 572 via email invitation links. While a precise response rate cannot be calculated, the completion rate for those who started the survey was 59.65%.

The full survey—comprised of 32 questions delivered with a mixed-method approach (i.e. open-ended and closed)—asked participants a range of questions related to their understanding and involvement in science communication activities. The first 17 questions in the survey, addressing general science communication issues, were answered by all our participants (*N* = 584), while the remaining 15 focused on science communication in the context of social media. These 15 questions were answered only by those participants who indicated that they use social media for science communication purposes (*N* = 314).

For the study presented in this article, we focus on a subset of questions from the survey discussed above. The subset of questions align with the aims of this article to investigate personal science communication definitions, to assess the extent to which the proposed shift from deficit to participation is reflected in the goals scientists identify as important to their general science communication efforts, and to assess the goals scientists identify as important to their social media science communication efforts. In the following paragraphs, we discuss the questions selected and the methods employed to analyse them.

### Personal definitions of science communication

An open-ended question asking participants to define science communication in their own words was used to gain a broader understanding of our participants’ individual viewpoints (or frames of reference) on what science communication is. In answering this question, participants were instructed to use either sentences or keywords to tell us what their personal definition of science communication is.

To analyze the ways in which our respondents define science communication, we adopted semi-inductive, qualitative thematic analysis. A widely used method in qualitative research, thematic analysis is a method to identify, analyze, and report patterns found in data [78]. While thematic analysis can be conducted in both an inductive and a deductive manner, here, we refer to a semi-inductive approach because although the coding emerged from the responses given by our participants, the coding topics were partially informed by the coders’ theoretical and conceptual knowledge in the field of science communication. For instance, definitions highlighting the “communication of scientific facts to the non-scientific communities” were given the label ‘Transmission’, which aligns with the one-way, top-down communication process prescribed by the deficit model.

Following the thematic analysis phases described by Braun and Clarke [78], the first author (1) familiarized themselves with the data by reading and re-reading the 584 definitions provided by our respondents. Next, the first author (2) inductively generated initial codes across the entire data set. This second step in the analysis was conducted until no further codes were found to be emerging from the data. During this iterative stage, codes were developed and merged, and clear definitions were created for each of the codes. Each of the 584 science communication definitions was assigned one or more of these codes, based on the types of communication the definition describes. A single code was not applied multiple times to the same definition. Once no new codes were found to emerge from the data, the first author proceeded to (3) collate the 30 codes that emerged from the data into six potential themes, (4) review the themes and their relation to the codes, and (5) name and refine the specifics of each theme. All the themes and codes, together with their definitions and in vivo examples, were collected into a codebook. Using this codebook, the second author also coded 100% of the data. Because the two authors coded the entire data set and because predefined quotations were not used for coding, we report Holsti’s index [79]. The inter-coder agreement between the two coders was 84.9%. 5.9% (35) of the definitions provided by our respondents were either too short or ambiguously formulated so that they could not be coded.

Lastly, the two authors discussed and reviewed the initial themes to insure clear and identifiable distinctions between them as well as meaningful coherence for each theme. To further confirm the reliability of the themes, Holsti’s index was calculated for each of them: Type of communication (87.7%), Audience (93.2%), Content (87.0%), Media (96.4%), Goals (77.2%), and Impact (79.2%). After this review, all the six themes initially identified were kept and included in our analysis.

### General science communication goals and models

To assess the goals respondents find most important to their science communication efforts and how these goals align with the conceptualisations proposed by the deficit, dialogue and participation models, we used a 28 item scale. Participants were asked to rate the importance of the 28 items to their science communication goals on a 5-point Likert-type scale, ranging from 1 = ‘Not important at all.” to 5 = “Extremely important.” This question appeared in the survey after respondents were asked to defined science communication in their own words.

The 28 items in our scale are based on the extensive literature review published by Metcalfe [30]. In her work, Metcalfe [30] used this 28 item scale with similar aims to ours: to assess theorised models against real-life science communication practice. However, her work, centred on the specific context of a 2012 Australian audit and 515 science engagement activities, found that most engagement activities had objectives that reflected a mix of deficit and dialogue activities and a lack of participatory activities in Australia [30]. Rather than focusing on activities, in our work, we will employ the 28-item scale to assess the goals our respondents find most important to their science communication efforts. Based on the literature review conducted by Metcalfe [30], each of the 28 items in the scale (listed in Table 1) align to one of the three science communication models as follows: goals 1 to 14 align with the deficit model, goals 15 to 20 align with the dialogue model (15-20), and goals 21 to 28 align with the the participation model. To ensure that the 28 items form a reliable scale, both as a whole and as three separate scales aligned with each of the three science communication models, Cronbach’s alphas were calculated for each. We find that the 28 items list as a whole (Cronbach’s *α* = .925) and also divided according to the three models form reliable scales (see Table 1).

**Table 1:** General science communication goals and models.

Deficit model goals (Cronbach’s *α* = .848)	Dialogue model goals (Cronbach’s *α* = .817)	Participation model goals (Cronbach’s *α* = .868)
1.To raise awareness	15.To be or to make science/scientists more accessible	21.To participate in a research endeavour with scientists
2.To transfer information	16.To find out public opinion or about audience needs	22.To get lay people involved in gathering data/doing research
3.To correct misunderstandings or misperceptions	17.To gain lay knowledge	23.To participate with other interests to influence the culture of science in society
4.To promote or gain support for science/scientists	18.To debate/discuss scientific/technological issues	24.To participate in democratic policymaking
5.To promote or gain funding for science	19.To help people to make decisions	25. To collectively learn, reflect, solve problems
6.To promote a particular scientific institution or organisation	20.To make connections between people, including between disciplines	26.To shape the agenda of science
7.To promote science as a career		27.To co-produce new knowledge/products
8.To inspire, build excitement, generate interest in science		28.To critically reflect on science and its institutions
9.To explain or increase understanding		
10.To educate or increase learning		
11.To respond to people’s interest in science		
12.To address people’s concerns about science and increase trust in science and scientists		
13.To influence people’s attitudes		
14.To influence people’s behaviour		

To verify if the 28-items in our scale align to the three science communication models, as proposed by the literature review conducted by Metcalfe [30], we used factor analysis. More specifically, we employed confirmatory factor analysis (CFA), a statistical technique used to verify that the theory-postulated relationships between observed variables and their underlying latent constructs exist [80]. The CFA was conducted using AMOS (version 26.0.0). First, we confirmed the 28 items in our scale are suitable for factor analysis through a Bartlett test (*χ*^2^(378) = 7762.743, *p* = < .000) and the Kaiser–Meyer–Olkin measure of sampling adequacy (KMO = .913). Next, factor loadings were assessed for each of the 28 items and three items with factor loadings less than the acceptable threshold of 0.30 [81] were removed from the analysis. Thus, from the initial 28 items presented in Table 1, items 6, 11 and 21 were removed from further analysis, which resulted in a final scale of 25 items. Strong factor loadings were found across the remaining 25 items, ranging from.42 to.71. Next, we evaluated the three-factor model fit using the relative Chi-square (*χ*^2^/*df*) [82], the comparative fit index (CFI) [83], the goodness of fit index (GFI) [84], the standardized root mean square residual (SRMR) [85], and the root mean square error of approximation index (RMSEA) [86].

Our results confirm the unidimensionality of each construct in our model and indicate that the measurement structure of three factors (Deficit, Dialogue, and Participation) and 25 items produced good fit statistics (see Table 2). As the sample size of our study is relatively large (*N* = 584), it is within expectation that the model did not pass the *χ*^2^ test (*p* < .05) [87–89]. Lastly, we found that even after the removal of the 3 items, the remaining 25 items form reliable scales, both as a whole (Cronbach’s *α* = .918) and as three separate scales aligned with each of the three science communication models: Deficit model Cronbach’s *α* = .830, Dialogue model Cronbach’s *α* = .817, and Participation model Cronbach’s *α* = .855.

**Table 2:** Confirmatory factor analysis results for a three-factor model of 25 general science communication goals (*N* = 584).

Indicators	Parameter estimates	Acceptance values
*χ*^2^/*df*	3.618	< 2–5
CFI	.901	>.90
GFI	.885	>.80
SRMR	.059	< .08
RMSEA	.067	< .08
*χ*^2^ = 889.973; *df* = 246; *p* = .000		

In our Results section, we explore these 25 general science communication goals individually (i.e., based on their item means across all respondents) as well as partitioned according to the three science communication models they represent, as confirmed by the factor analysis reported above. For the latter, we computed two aggregate variables for each of the three latent variables/models (Deficit, Dialogue, and Participation): 1) the unit weighted scale means and 2) factor scores using factor score weights produced by the CFA, normalized to allow for comparison across models [90–92]. While the bulk of the statistical analysis in this paper will focus on factor scores, the results from the scale means are also reported; these have been argued to hold complementary utility that is less dependent on sample characteristics (e.g., replicability of findings across studies [93]). After we computed the pairs of variables for each respondent and communication model, Shapiro-Wilk tests [94, 95] showed a significant departure from normality for all of our variables (see Table 3). Due to the variables’ non-normal distribution, and because the variables from each model are based on related samples, we used Wilcoxon signed-rank tests to determine whether the differences in how our respondents rate goals of the three science communication models are statistically significant [96, 97]. As a non-parametric statistical test that compares two paired groups, the Wilcoxon signed-rank can be used as an alternative to the t-test when the population data does not follow a normal distribution [97].

**Table 3:** Shapiro-Wilk tests—General science communication models.

Shapiro-Wilk					
		Means		Factor scores	
	df	W	*p*	W	*p*
Deficit	584	.978	0.000	.979	0.000
Dialogue	584	.969	0.000	.982	0.000
Participation	584	.982	0.000	.984	0.000

### Social media science communication goals and models

Additionally, using Metcalfe’s [30] scale, we selected nine of the 28 general science communication goals that were most suited to the particulars of social media science communication—three goals for each of the three models discussed—to create a scale for social media science communication goals (Cronbach’s *α* = .846). Although the items in this scale were specifically worded for the social media context, the question asked respondents to rate the importance of these items for their social media science communication activities, just as in the case of their general science communication goals. The nine items aligned to the deficit model (1-3), the dialogue model (4-6), and the participation model (7-9) and they form reliable scales (see Table 4).

**Table 4:** Social media science communication goals and models.

Deficit model goals (Cronbach’s *α* = .740)	Dialogue model goals (Cronbach’s *α* = .740)	Participation model goals (Cronbach’s *α* = .692)
1.Raise awareness	4.Make science/scientists more accessible	7.Help get lay people involved in gathering data/doing research
2.Transfer science information	5.Help scientists gain lay knowledge	8.Help co-produce new knowledge/products
3.Correct misunderstandings and/or misperceptions	6.Help scientists find out public opinion or about audience needs	9.Help critically reflect on science and its institutions

Just as in the case of the general science communication goals, we ran confirmatory factor analysis to assess whether the nine items in our scale align with the three science communication models, as proposed by Metcalfe’s literature review [30]. We first confirmed that the 9 items in our scale are suitable for factor analysis through a Bartlett test (*χ*^2^(36) = 963.682, *p* = < .000) and the Kaiser–Meyer–Olkin measure of sampling adequacy (KMO = .872). Next, and unlike in the case of the general science communication goals, no items were removed from the three-factor model due to strong factor loadings, ranging from.60 to.76. Using the same model-fit evaluation measurements (*χ*^2^/*df*, CFI, GFI, SRMR, and RMSEA), we confirmed the unidimensionality of each construct in our model. Hence, a three factor model (SM Deficit, SM Dialogue, and SM Participation) with 9 items produced good fit statistics (see Table 5). Because our sample size is relatively large, the model did not pass the *χ*^2^ test, as models based on large sample sizes have a higher tendency to be rejected [87–89].

**Table 5:** Confirmatory factor analysis results for a three-factor model of 9 social media science communication goals (*N* = 314).

Indicators	Parameter estimates	Acceptance values
*χ*^2^/*df*	3.188	< 2–5
CFI	.949	>.90
GFI	.954	>.80
SRMR	.054	< .08
RMSEA	.064	< .08
*χ*^2^ = 70.132; *df* = 22; *p* = .000		

Just as we did for the general science communication models, in our results section, we explored the nine social media goals individually, but we also computed latent variables for each model, using the factor score weights as well as the unit weighted means of each scale. An exploration of these three new pairs of variables (for each SM Deficit, SM Dialogue and SM Participation) also shows a statistically significant departure from normality, as shown by the Shapiro-Wilk tests in Table 6.

**Table 6:** Shapiro-Wilk tests—Social media science communication models.

	Shapiro-Wilk				
		Means		Factor scores	
	df	W	*p*	W	*p*
SM Deficit	314	.929	0.000	.955	0.000
SM Dialogue	314	.945	0.000	.970	0.000
SM Participation	314	.972	0.000	.981	0.000

## Results

Before describing our findings, let us first describe the demographics and characteristics of our survey respondents. 54.6% of our respondents identified as female, 43.2% as male, and 0.9% as non-binary/third gender, while 1.4% preferred not to say. Most of the respondents in our data set held a PhD (58.6%) or a Masters’ (38.9%) degree, with 77.1% being employed under a full time contract. Interestingly, as per Table 7, the majority of respondents in our sample were early-career researchers (including 37.8% Ph.D. candidates and 11.8% Postdoctoral researchers). We further elaborate on the implications of this finding in our discussion.

**Table 7:** Demographics (*N* = 584).

	N	%
**Gender**		
Female	319	54.6
Male	252	43.2
Prefer not to say	8	1.4
Non-binary / third gender	5	0.9
**Level of education**		
Doctorate	342	58.6
Master’s degree	227	38.9
Bachelor’s degree (Dutch WO)	7	1.2
Other	5	0.9
Higher professional education (Dutch HBO)	3	0.5
**Current academic position**		
Ph.D. Candidate	221	37.8
Assistant Professor	94	16.1
Postdoctoral Researcher	69	11.8
Full Professor	52	8.9
Associate Professor	45	7.7
Researcher	29	5.0
Lecturer	28	4.8
Other	26	4.5
Senior Researcher	8	1.4
Independent Researcher	7	1.2
Research Assistant	3	0.5
Student Teaching/Research Assistant	2	0.3
**Current employment**		
Employed full time	450	77.1
Employed part time	97	16.6
Student	15	2.6
Retired	14	2.4
Unemployed looking for work	7	1.2
Unemployed not looking for work	1	0.2

In the following subsections, we discuss our findings regarding our respondents’ personal science communication definitions, their general science communication goals, their social media science communication goals, and how these goals align with the three science communication models discussed in the introduction of this paper, namely the deficit, dialogue, and participation model.

### Personal definitions of science communication

The thematic content analysis conducted on the 584 definitions provided by our survey respondents resulted in 30 codes and 6 themes. These codes and themes highlight the various science communication aspects, dimensions, and perspectives our respondents highlighted in their definitions. In Table 8, we show our codes, their occurrence frequency, and an example for each code as well as the themes emerging from collating our codes. Here, we should note that a code was applied to each definition provided by our respondents only once. In Table 8, the codes are organized by theme and ordered by frequency.

**Table 8:** Qualitative analysis results: Themes, codes and examples.

Theme	Code	Freq.	Example
**Type of comm**	Transmission/transfer	372	“Disseminating academic research and the insights of this research”
	Translation	161	“Science communication is about translating scientific insights to another (not necessarily wider) audience.”
	Discussion/debate	21	“Discussing and dispersing ideas about our research and results.”
	Exchange	21	“When scientists collaborate for a project or exchange ideas on a project.”
	Interaction	18	“Interactions between scientists and non-scientists about the process and results of research.”
	Engagement	17	“It is to convey results or plans of science work with the objective to engage audiences”
	Participatory	10	“Science communication ensures the interaction, engagement, and participation of the society with science and scientists.”
**Audience**	Non-academic audience	363	“Communication of scientific results to the public”
	Academic audience	136	“The communication of research findings to academics”
	Interested audience	7	“To talk about your curiosity-driven research hypotheses and ideas to an interested audience”
	Uninformed audience	5	“Sharing of research outcomes with the purposes of informing the uninformed”
**Content**	Research results	250	“Disseminating the results of research conducted via scientific methods to the general public.”
	Research process	66	“Interactions between scientists and non-scientists about the process and results of research.”
	Research relevance	41	“Communication about the need for, relevance, findings, and use made of scientific research”
	Research methods	32	“Sharing results or methods of my research with my colleagues, other stakeholders and the general public.”
**Media**	Traditional Media	34	“Explaining research to others (e.g public), which can be in many forms (e.g. via newsitems, presentations, publications etc.)”
	Digital & Social media	23	“Disseminating scientific results to the general public and non-academic stakeholders through social media, publications, events, and interviews”
**Goals**	Making science accessible	32	“Making science accessible and understandable for the broader public”
	Educational	24	“The goal could be to inform, to educate, to raise awareness and to make them curious.”
	Raising awareness	23	“Raise awareness, spread the truth and facts, attract people to think”
	Enhancing interest in science	21	“Communicating research to […] audience to enhance their interest in your project and science in general”
	Improve understanding	9	“The presentation of findings, in a that seeks to improve public understanding of the sciences”
	Bridge science and practice	8	“Science communication acts as a bridge (mediator) between research and practice or science and everyday people.”
	Popularizating science	7	“This can take many forms, from popularizing pieces, to interviews, to participation in art projects.”
	Spreading scientific facts	7	“Communication of scientific facts to the non-scientific communities.”
	Influence audiences	6	“Communicating research to a wide, non-specialised, audience to enhance their interest in your project and science in general”
	Inspire audiences	6	“Make people understand why science is fun, important, and useful.”
**Impact**	General Impact	15	“Creating impact with knowledge.”
	Societal impact	11	“Explain intricate research and its impact on society”
	Practical impact	4	“Explaining and promoting science for a lay audience, including theory, empirical research and practical implementations”

Overall, our respondents highlighted six main dimensions of science communication, as shown by the themes that emerged from their definitions. These themes center on the type of communication (*N* = 620), the audience addressed (*N* = 511), the content of the communication (*N* = 389), the goals to be achieved (*N* = 148), the medium through which the communication takes place (*N* = 57), and the impact of science communication (*N* = 30). We will discuss each of these themes and their most prominent codes individually.

#### Type of communication

Within the type of communication theme, the most prominent dimension we uncovered during our coding was that of information transmission/transfer. As shown in Table 8, most definitions (63.7%) made mention of science communication as an information transmission process, using terminology such as disseminating, transmitting, or transferring scientific knowledge or information to an audience. As we will discuss later, this process of transmission was highlighted regardless of the type of audience addressed. The second most prominent emerging dimension was that of translating scientific information (27.57%), while dimensions related to more dialogic, engagement, or participatory dimensions of science communication emerged in a very limited number of definitions. Thus, the focus on information transmission and translation seems to suggest that most of our respondents consider science communication to be a one-way process of information diffusion, from the knowledgeable scientist to the uninformed audience.

#### Audience

Our qualitative analysis showed that the next most prominent theme our respondents focused on when defining science communication is the audience to be addressed. While most respondents mentioned a lay or non-academic audience (62.16%), a good proportion also included academic audiences in their definitions (23.29%), while very few made reference to interested (1.20%) or uninformed audiences (0.86%). In sum, when defining science communication, our respondents identify academic as well as non-academic stakeholders as main audiences for their science communication efforts. 111 (19.01%) definitions included both, academic and non-academic audiences. As we will discuss later, these definitions may be indicative of a dichotomy, a separation between scientists and other members of the public, which has further implications.

#### Content

The third most common theme emerging from these personal definitions of science communication was the content of their communication efforts. Here, most respondents emphasized research findings or results as the most common dimension of their definition (42.81%). Fewer respondents made mention of involving their audience in the process of science (11.30%) or of communicating the relevance (7.02%) and methods (5.48%) of scientific research. Considering our earlier finding—that most personal definitions we investigated focused on a one-way, information transmission model of science communication—it then come as no surprise that the most predominant content mentioned was research results. This would suggest that our respondents look at science communication as a process of transferring a ready-made product of science (results) to their audiences, whether academic or not.

#### Goals

Our analysis also uncovered a number of goals highlighted in the science communication definitions provided by our respondents. The most prominent of these goals was that of making science more accessible (5.48%), educating their audience (4.11%), raising awareness about science and scientific topics (3.94%), and enhancing interest in science (3.60%). The other six goals emerging in our analysis (see Table 8) were far less frequently mentioned by our respondents. As we will discuss later in this paper, most of these goals are very much in line with the deficit model of science communication, with the exception of the goal of making science more accessible, an inherently dialogue-oriented goal [30].

#### Media

The fifth theme uncovered by our analysis was that of the medium through which science communication takes place from the perspective of our respondents. Here, 5.82% definitions mentioned traditional media (e.g., newspapers, radio, TV etc.) as the primary channel for science communication, while 3.94% made mention of digital and social media (e.g., blogs, Twitter, podcasts) as science communication platforms. This suggests that among those who include a means or channel of communication in their definitions, the majority made reference to more traditional forms of media, and fewer see digital and social media as a science communication venue.

#### Impact

The last and least prominent theme that emerged from our analysis was that of impact. The definitions formulated by our respondents included communicating about the general (2.57%), societal (1.88%) and practical (0.68%) impact of their research.

To summarize, the qualitative thematic analysis of our respondents’ personal science communication definitions aimed at creating a broader understanding of what science communication is, from the perspective of those who are closest to the scene. While not trying to discount the very few personal definitions that have included aspects of dialogue-based and participatory science communication, our results suggest that the majority of these definitions are centered around a deficit way of thinking by emphasizing a one-way, transmission model of communication that aims to transfer and translate research outcomes and its impacts to a non-academic audience, with the purpose of making science more accessible, of educating audiences, and/or of raising awareness about science.

Having investigated how our participants define science communication, in the next sections of our paper we quantitatively analyze our respondent’s general science communication goals, as well as the goals they find most important when specifically asked about social media science communication. Furthermore, we discuss how these goals aligned with the deficit, dialogue, and participation models of science communication.

### Science communication models

**Fig 1:**
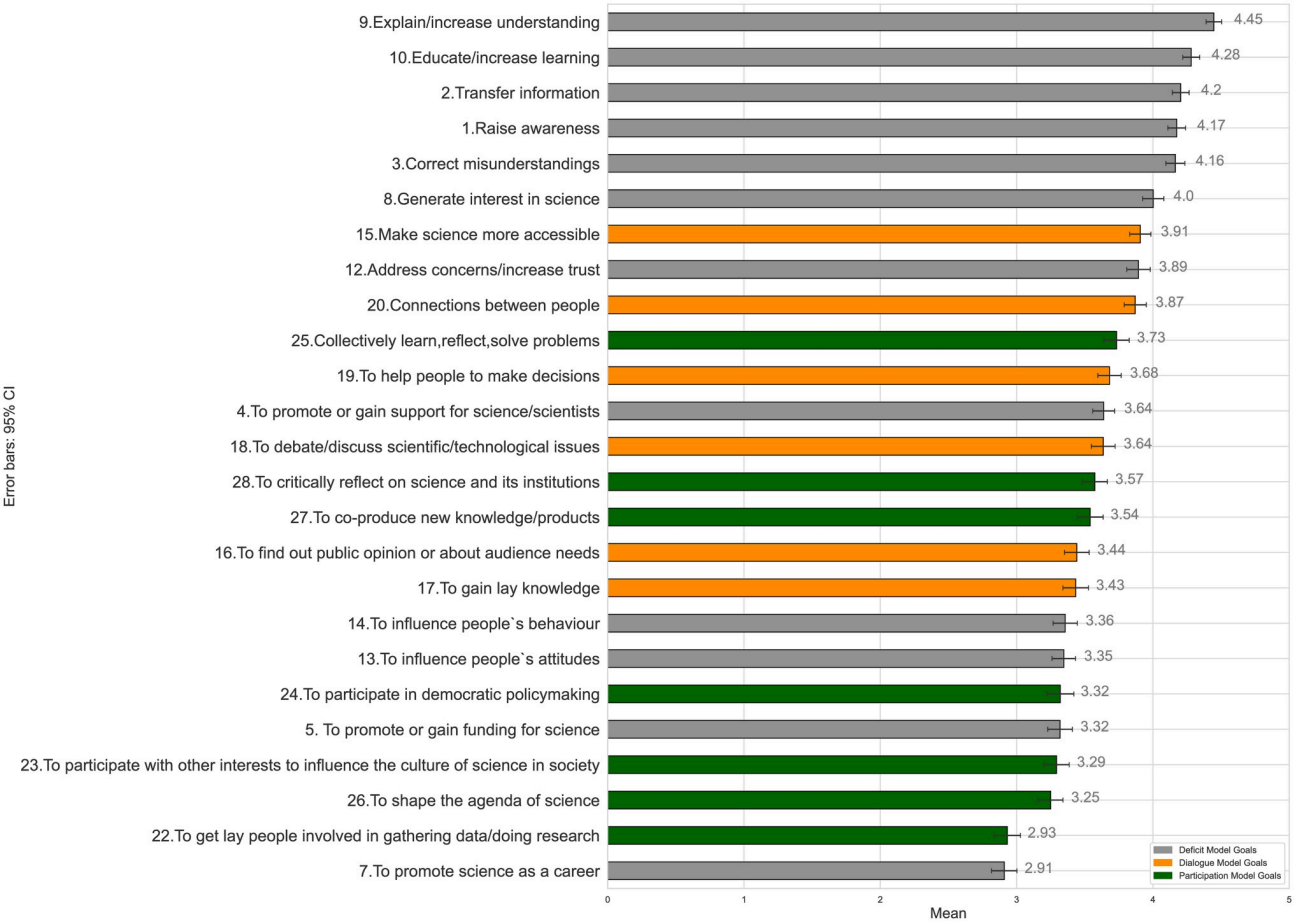
General science communication goals ordered by mean (*N* = 584).

We start the description of our quantitative analysis results with the ways in which our respondents rated the importance of the 25 general science communication goals and the science communication models they represent. Our results indicate that, on average, our respondents rated deficit model goals as more important than dialogue model goals and participatory model goals. In Fig 1, we show the means of each of the 25 goals, in descending order and colored by the communication model they align with. The highest rated goals in our scale were deficit model goals. Based on the rating of our respondents, increasing understanding of science, educating, transferring information, raising awareness, and correcting misunderstandings or misconceptions were the most important goals to their science communication efforts. This particular finding resonates with our qualitative analysis findings, in that it seems to indicate that a deficit way of thinking was the predominant approach to science communication among our respondents.

The means of each model—as already hinted at by our qualitative results and by looking at the highest rated goals—indicate that our respondents did indeed rate deficit model (*M* = 3.81) and dialogue model goals (*M* = 3.66) as more important than participation model goals (*M* = 3.38). A Wilcoxon signed-rank test of the factor scores further confirm that our participants rated Deficit model goals higher than Dialogue model goals and that this difference is statistically significant: *T* = 133157;*z* = −12.530;*p* < .001. We also find that on average, our participants rated Deficit model goals (*T* = 138057;*z* = −13.757;*p* < .001) and Dialogue model goals (*T* = 139912.50;*z* = −14.221;*p* < 001) significantly higher than Participation model goals. The same significance levels are also found between pairs of models when testing the scale means rather than the factor scores (*p* < .001 for each pair). Thus, we can infer that in terms of science communication goals, our respondents focused more on a Deficit and Dialogue perspective, rather than a participatory approach.

Analyzing the personal definitions and the goals our respondents find most important to their science communication activities and efforts has thus far provided a relatively straightforward portrayal of mostly deficit or dialogue oriented perspectives, with participatory approaches underrepresented or entirely lacking. However, considering the emergence of social media and the promises of such digital channels to be the ideal environment for two-way and multi-vocal science conversations, we move on to investigate whether those actively using social media for their science communication efforts may approach science communication from a more participatory oriented perspective.

First, we note that from our 584 our respondents, only 314 (53.77%) reported that they use social media for science communication. Our 314 participants who said they use social media for science communication reported that they used LinkedIn (38.9%), Twitter (33.4%), Facebook (15.8%), and YouTube (0.9%) for science communication in the past 12 months, and also other less frequently used platforms (e.g., Instagram). All of these most commonly used platforms among our respondents offer affordances that can be used to enter a dialogue with or involve already-engaged audiences into participatory science communication activities. Thus, in the next paragraphs, we assess what type of goals those participants who use social media for science communication rated as most important.

**Fig 2:**
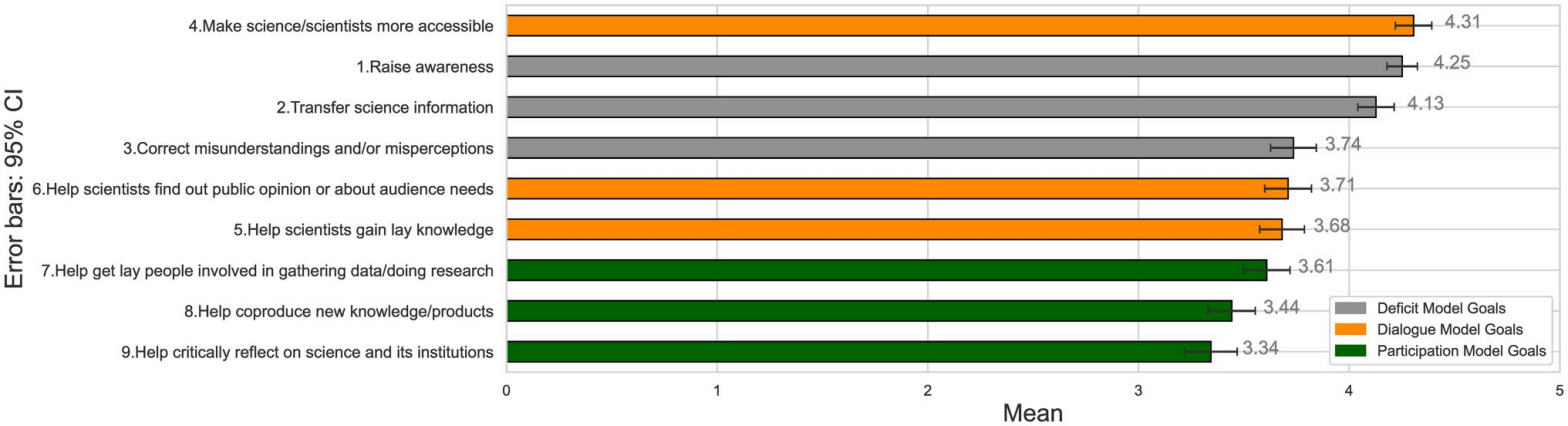
Social media science communication goals ordered by mean (*N* = 314).

When looking at how our respondents rated their agreement to the 9 statements related to social media science communication goals (see Fig 2), we found that the highest rated goal is a dialogue model one, namely the goal of making science/scientists more accessible. Unlike in the case of general science communication, where respondents rated a deficit model goal as the most important, we see that when specifically focusing on social media, participants rated a dialogue model goal as most important. This would seem to indicate that perhaps in the context of social media, respondents moved from the deficit view to a more dialogue oriented one. However, the next two highest rated goals are related to raising awareness and transferring science information, two inherently deficit model oriented goals. Furthermore, we note that overall the highest rated goals (based on their means) were those of the Deficit model (*M* = 4.04) followed by Dialogue model (*M* = 3.90), while Participation model goals were rated lowest (*M* = 3.47). To further confirm the significance of this finding, we performed Wilcoxon signed-rank tests on the factor scores of our model variables. The results confirm that, on average, our participants rated Deficit model goals significantly higher than Dialogue model goals, *T* = 31957.00, *z* = −8.025, *p* < .001, and significantly higher than Participation model goals, *T* = 36779, *z* = −11.452, *p* < .001. The tests also show that, on average, Dialogue model goals were rated significantly higher than Participation model goals, *T* = 38018.50, *z* = −12.332, *p* < .001. Significance levels are found to be similar when using means instead of factor scores as well. Thus, when specifically asked about their science communication goals in the context of social media, and even though social media promises to be an ideal environment for dialogue and participation, our respondents’ focus remained mostly on the deficit-oriented perspective.

Additionally, our analysis found statistically significant differences in how respondents who reported using social media for science communication (*N* = 314) rate their *general* science communication goals, when compared to those who reported not using social media for science communication (*N* = 270). Overall, the factor score means listed in Table 9 indicate that social media users rated goals associated with all three models higher than non-users, and the Mann-Whitney U tests for each pair further confirm that these differences are statistically significant. Thus, in all cases, social media users rated goals significantly higher than non-users. When using scale means and Mann-Whitney U tests, we find similar significance, with the exception of the Participation model goals (*p* = .124).

**Table 9:** Mann-Whitney U tests.

	Means (Factor scores means)		Factor scores	
	SM users	SM non-users	*U*	*z*
Deficit	3.85 (3.87)	3.75 (3.76)	38364.50	-1.980*
Dialogue	3.75 (3.69)	3.55 (3.54)	37875.50	-2.221*
Participation	3.43 (3.63)	3.31 (3.48)	38248.50	-2.037*

*p < .05

This particular part of our analysis showed that when comparing between social media users and non-users, we found important differences. Interestingly, social media users rated their general science communication goals higher than non-users, perhaps indicative of higher activity among social media users in communicating science.

## Conclusion

Using a mixed-methods approach, this article set to uncover scientists’ personal science communication definitions; the goals they find important to their science communication efforts; the alignment of these goals to the deficit, dialogue, and participation models of science communication; and whether a deficit-model way of thinking remains dominant in social media science communication efforts. In the following paragraphs, we discuss our findings, starting with the qualitative thematic analysis results, followed by our quantitative findings and in the context of previous research. We discuss whether the highly popularised—and theorised—rhetorical shift from deficit to participation is reflected by our results, and we argue that we should be cautious of recent claims that we live in a new era of dialogue, transparency, and participation in the realm of science communication.

### Personal definitions

The field of science communication has grown considerably over the past decade, and so have the number scientific writings on what science communication is. As an inherently multidisciplinary field—concerned with answering questions related to science and society, science in the media, and the role of science journalists—science communication research has produced many different theoretisations, models, and definitions of what science communication is and how it should be practiced. The lack of clarity in defining science communication, and the absence of extensive research into the perspectives of those at the centre of science communication, have prompted us to investigate scientists’ personal definitions in order to better understand how science communicators themselves define it. Our analysis of the 584 definitions provided by our survey respondents showed that, for scientists, there were six dimensions of science communication: the type of communication employed, the audience addressed, the content of the communication, the goals to be achieved, the medium through which the communication takes place, and the impact of science communication. Based on these six dimensions, most of our respondents define science communication as:

A one-way process of transmission and translation of scientific results and their impacts towards a lay audience, via mostly traditional media channels, with the goals of making science more accessible, of educating audiences, and of raising awareness about science.

This definition suggests an alignment between our results and those of previous studies that found scientists tend to favour one-way communication with the public via the media, viewing engagement as chiefly about dissemination of scientific knowledge [23]. This approach to science communication assumes that the scientific information transmitted will be interpreted in similar ways by all the members of the audience and that the transmission of scientific knowledge will generate understanding and support of science [36]. Also, this one-sided, informative, sender-receiver communication style draws an intellectual boundary between scientists and the public, portraying the public as a passive recipient of information [63]. Furthermore, the focus on translation of scientific information suggests the scientist needs to simplify complex information so that it can become accessible to the scientifically uneducated. As previous research has shown, scientists are often aware of the need to present material differently between different discourses, audiences, and situations, yet when specifically moving from an academic to a non-academic audience, “their conception of the changes entailed […] were conceived only as ones of simplification.” [98, p. 444]. While the process of engaging with non-academic audiences does often entail the need for translation or simplification, what is ignored when focusing only on simplification is the need to include political and ethical implications, aspects which are often excluded form scientific analysis but highly relevant and legitimate in non-academic contexts.

The focus of this definition on mostly non-academic audiences, as well as the distinction some respondents made between academic and non-academic audiences, suggests a dichotomy of “us-them.” As previous research has already suggested, this separation between scientists and other members of the public may indicate that scientists do not see non-academics as part of the scientific dialogue or debate [99] and that they see the public as homogeneous [23]. Viewing their audiences as a uniform group of non-experts leaves little room for “any relevant expertise outside of the scientific community, or for any intermediate degrees of scientific knowledge or understanding.” [98, p. 437] However, in today’s knowledge societies, the boundary between experts and non-experts is more fluid than ever [100], with publics asking to participate in the processes of knowledge production more and more and asking for that process to be made more transparent and available for public inspection [101]. The continued adoption of a simplistic contrast between science and the public as two completely separate and ‘antagonistic’ entities hinders a true shift towards more democratic public engagement [63].

Thus far, the emphasis on transmission and translation and the clear distinction between academic and non-academic audiences, together with the goals to educate and raise awareness, seem to indicate a mostly deficit driven approach to science communication among our respondents. The deficit way of thinking, or the deficit model of science communication, is guided by the belief that the public has a deficit in scientific knowledge that the scientist needs to fill through dissemination or education [38]. However, the science communication definition formulated by our respondents also includes elements of a more dialogue oriented perspective, by formulating the goal of making science more accessible. The dialogue way of thinking, or the dialogue model, centres on science communication as a two-way flow of information from expert to layperson and vice versa. While some may see the goal of making science more accessible as an inherently deficit oriented goal, we argue that this goal is the first step towards a dialogue model perspective. Making science and scientists more accessible creates opportunities for dialogue between scientists and the public to discuss scientific issues and discover public opinion [30, 102]. We do note that other elements of the dialogue model were not at all prominent in the science communication definitions we analysed.

### Science communication goals

The next aim of our investigation was to understand the goals scientists rate as most important, both in a general science communication context as well as a social media context. Scientists’ specific goals are crucial in understanding not only what scholars want to get out of the time and resources they invest into communication, but also in understanding their approach to science communication. When assessing the goals our respondents found to be most important for their general science communication efforts—and how these goals reflect the theorised science communication models—we found similar results as with their science communication definitions. Our respondents rated goals aligned with the deficit and dialogue models of science communication to be the most important.

In the context of social media, we found that our respondents’ focus remains mostly on the deficit-oriented perspective, which seems to be in direct contrast with the promises of social media as interactional spaces, ideal for two-way and multi-vocal science communication activities [39]. Perhaps, as Weller [69] noted, this is a result of the reluctance scholars have shown in changing established science communication patterns in response to social media. However, as other studies have also found, focusing on achieving mostly deficit oriented goals in the context of social media leads to an underutilization of social media’s potential to create true dialogues and engagement with their audiences [70–72].

## Discussion

Over the last two decades, scientific as well as funding and policy initiatives, in many countries, have shifted their rhetoric to move away from deficit ‘public awareness of science,’ and ‘science and society’ towards ‘citizen engagement,’ ‘dialogue,’ and ‘science *in* society.’ This change in terminology was suggested to be indicative of a transition from a deficit perspective on science communication, towards dialogue and participation. However, many have deemed this shift to be highly implausible over such a short period of time [37, 46] and that most science communication initiatives are still guided by the misconception that deficits in public knowledge are the driving force behind conflict over science [36]. Even those outreach projects and activities that claimed to follow other theoretical approaches have been shown to use the deficit model approach as a backbone [29].

With this study we add to research evidence that has questioned the degree to which science communicators have truly adopted the goals of this proposed shift towards dialogue and participatory science communication [50, 51, 103, 104]. Our results, though limited to scientists in the Netherlands, showed a predominant focus on the deficit model of science communication, with limited elements of a more dialogue centered approach and almost no emphasis on participatory approaches.

This narrow emphasis of the deficit approach does not allow for the recognition that scientific knowledge is not the only factor influencing how individuals reach judgments. Decades of research have shown not only that science literacy plays a very limited role in shaping public perceptions and decisions, but also that people with limited education can quickly come to understand highly complex technical information [42, 105]. Furthermore, simply sharing science information in a unidirectional fashion is not an adequate way of increasing science knowledge or changing attitudes about science [106, 107]. Taking a deficit approach to science communication presents a major obstacle for reflective exchange and mutual learning, and ultimately for the transition towards more democratic forms of public involvement [63].

As noted in our results and conclusion, our data also showed evidence of more dialogue-driven approaches among our respondents. This dialogue model perspective was evident in both the ways in which respondents defined science communication and in the general science communication goals they rated as most important. However, while the dialogue perspective is driven by aims of engaging in two-way conversations, this dimension was not prominent in our data. Rather, our participants highlighted the importance of making science and scientists more accessible. Because our respondents consider being and making science more accessible as important goals, perhaps a transition away from dissemination and towards an exchange of perspectives between scientists and the public is not out of the question. As Metcalfe [30] also argues, making science and scientists more accessible may make it easier for the public to engage with science, and it could be considered a perquisite for more sophisticated dialogue activities that have the potential to open up productive and surprising discussions [108].

Arguably, thus far, our discussion may read as yet another pejorative scholarly discussion about the deficit way of thinking that argues for a clear separation between deficit, dialogue, and participatory science communication approaches. This is not our intention and such a discussion would be rather unhelpful for the development of science communication. Rather, the few elements of dialogue-driven science communication approaches, together with the prominent deficit elements uncovered by our study, lead us to argue that scientists do not adhere to a single model of science communication and that a clear separation between these models is not realistic. Instead, as other studies have also found, a combination of goals and practices—inherent to different models—govern science communication efforts and perspectives [30, 52].

What is of great concern, however, is that participatory aspects of science communication were nonexistent in most of the definitions formulated by our participants and that participatory goals were invariably rated the lowest, whether in a general or a social media context. Here, we are not trying to argue that deficit or dialogue model based activities are inherently inferior, nor do we want to suggest that such activities should be entirely abandoned. Rather, we would like to emphasise the importance of true engagement with various publics, especially when dealing with the complexities of current contemporary issues. Even when deficit model approaches are at the backbone of such science communication activities, participatory elements can be invaluable tools for reflecting upon science and knowledge, creating new knowledge, and most importantly, placing science in a wider social context [10, 38].

This lack of focus on participatory goals among our respondents is especially problematic considering the demands of contemporary knowledge societies and in the face of fast-paced technological advancements. The rise of digital platforms and social media have created an entirely new ‘ecology’ of communication between science and the public [109]. In the past scientists and science organizations have relied on science journalists or professional communicators to interpret scientific information for the general public. While to a certain extent this still holds true today, digital media platforms have made it possible for scientists to communicate directly with publics [110]. This digital transformation of science communication comes with both positive and negative consequences. On the positive side, digital platforms can be used to facilitate broader involvement of citizens in science discussions and they can allow individuals to learn about science and to become involved in collective decision-making [111]. On the negative side, the abundance of information sources available to individuals and the prevalence of misinformation in digital media create an urgent need for science-society relationship and trust building [112].

While it may still be possible to communicate scientific findings in accordance with the traditional deficit model, it has become increasingly difficult to generate public trust in science on the basis of pure scientific testimony alone [100]. As Elam [100] states, in contemporary knowledge societies the challenges facing science communicators are no longer those of combating public ignorance, but rather those of creating “new forms of community around radical programmes of research still in the making.” (p. 251) Scientists need to re-frame their view of technology and digital platforms and consider their uses in more robust, relationship building activities [110].

The findings presented in this study are based on the results of a survey administered among scientists in the Netherlands. As it is with most surveys, and even though we made great efforts in disseminating and publicizing our study, only 584 complete responses were collected. Thus, our results and the subsequent discussion should be viewed in the context of a limited number of responses provided by scientists in the Netherlands.

Additionally, we saw that a large number of our respondents were early-career scientists (Ph.D. candidates and postdoctoral researchers). Previous studies discuss a shift in the culture surrounding more participatory approaches to science communication to be driven by early-career scientists [113]. However, our findings—although based on a sample in which early career scientists are over-represented—suggest an overall adherence to deficit model approaches, with some elements of dialogue. For a discussion on some of the factors (institutional and personal) that may impact the lack of more participatory oriented approaches to science communication, in the context of the United States, see [113, 114]. Moreover, while Howell and colleagues [115] found that both late and early career scientists held positive views on the role of social media in providing opportunities to engage with the public, our respondents’ focus nevertheless remained on deficit-oriented goals when asked about their social media science communication goals. While we can only speculate that perhaps the differences between the academic contexts of the Netherlands and of the United States might be the underlying cause of these differences, further research comparing academic contexts and science communication approaches among scientists (much like the one conducted by Kessler and colleagues [21]) could shed more light on the issue.

The demographics collected in our survey did not include information about the affiliations of our respondents. Although this particular choice in the survey design offered respondents more anonymity, our results cannot speak to differences between scientists affiliated with different universities in the Netherlands, nor can they make distinctions between scientists from different disciplines. Previous studies have found that scientists’ academic fields play a role in their approaches to science communication. For example, Burchell found that participatory approaches to science communication was considerably higher in fields like the arts, humanities, and social sciences compared to the STEM (Science, Technology, Engineering, and Mathematics) fields [116]. Similar results are also reported by Kessler and colleagues, with scientists from the humanities, social sciences, and life sciences being more likely to adhere to dialogue and participatory perspectives [21].

Our study builds further evidence that a deficit model approach to science communication is still very much present [73, 74, 99, 117], even in the face of increasing calls towards science communication approaches that foster dialogue and public participation [36]. Simis and colleagues [99] discuss four lines of reasoning for why the deficit way of thinking remains very much present in science communication. Their essay highlights the roles of scientists’ training in processing information in a rational manner, the lack of formal public communication training, viewing the public as “others”, and the fact that the deficit model works well for policy design as some of the most prominent reasons for the persistence of the deficit model.

Understanding that scientists are focused mostly on dissemination and translation of research results, with a willingness to make themselves and science more accessible, has important consequences for scientific organizations, the shaping of their long-term science communication strategies, the development of training programs for established scientists, and the inclusion of science communication curricula in graduate education programs. As Madden, Cacciatore, and Yeo [118] observe, without continued training in positive communication methods, grounded in social science research, “it is not surprising that scientists would follow the admittedly intuitive deficit model.” (p. 403) The importance of continued training in positive communication methods as well as the importance of engaging community members around scientific issues are also emphasized by Simis and colleagues [99] as ways of moving beyond knowledge deficit approaches.

Lastly, the abundance of science communication models proposed by many scholars over the years could not all be addressed in our work. For example, one established model we did not address is the contextual model [25]. This model is generally tied to particular audiences, focuses on particular context-dependant situations (e.g., locations), and highlights the ability of audiences to quickly become knowledgeable about relevant topics [29]. However, while this model highlights the importance of contextual factors in how individuals receive and process information, it nonetheless conceptualizes the same focus on the responses of individuals to scientific information, just as the deficit model does [119]. Another model that was not included in our investigation is the one described first by Scheufele [120] and later by Akin and Scheufele [121], namely the communication in context model. Also known as the science communication as political communication model, this expanded model of science communication takes into account the larger political contexts of science communication, acknowledges that scientists are just some of the many voices involved in scientific debates, and highlights the many science-related streams of information the public is exposed to. As Scheufele [120] argues, answering questions about emerging technologies will require “democratic decision making that draws on moral values, that weighs complex political options, and that includes debates about the ethical and legal aspects” (p.13590). Thus, it is reasonable to argue that such models can help us move beyond deficit perspectives and that they better fit with the realities of our contemporary societies and the complexities of current scientific advances. We encourage future studies to include such models in their investigations.

To conclude, science communication and public engagement can no longer be focused on persuading the public on the ‘facts’ of science, as we live in an era where most science debates are no longer small, localized events, controlled by scientists or science communicators. As such, and as Nisbet and Scheufele [36] also proposed, contemporary science communication and public engagement activities should move away from a deficit, top-down perspective, towards promoting conversations that “recognize, respect, and incorporate differences in knowledge, values, perspectives, and goals.” (p. 1777)
